# Supplementary material for: Causal Relationship between Aging and Anorexia Nervosa: A White-Matter-Microstructure-Mediated Mendelian Randomization Analysis
Source: Biomedicines. 2024 Aug 16;12(8):1874. doi: 10.3390/biomedicines12081874 (PMC11351342; doi:10.3390/biomedicines12081874)
Supplement: Supplementary file 1 [file biomedicines-12-01874-s001.zip › Supplementary Figure/Figure S4/Singe SNP plot of MR analysis for TL to ubm-b-1983.pdf]

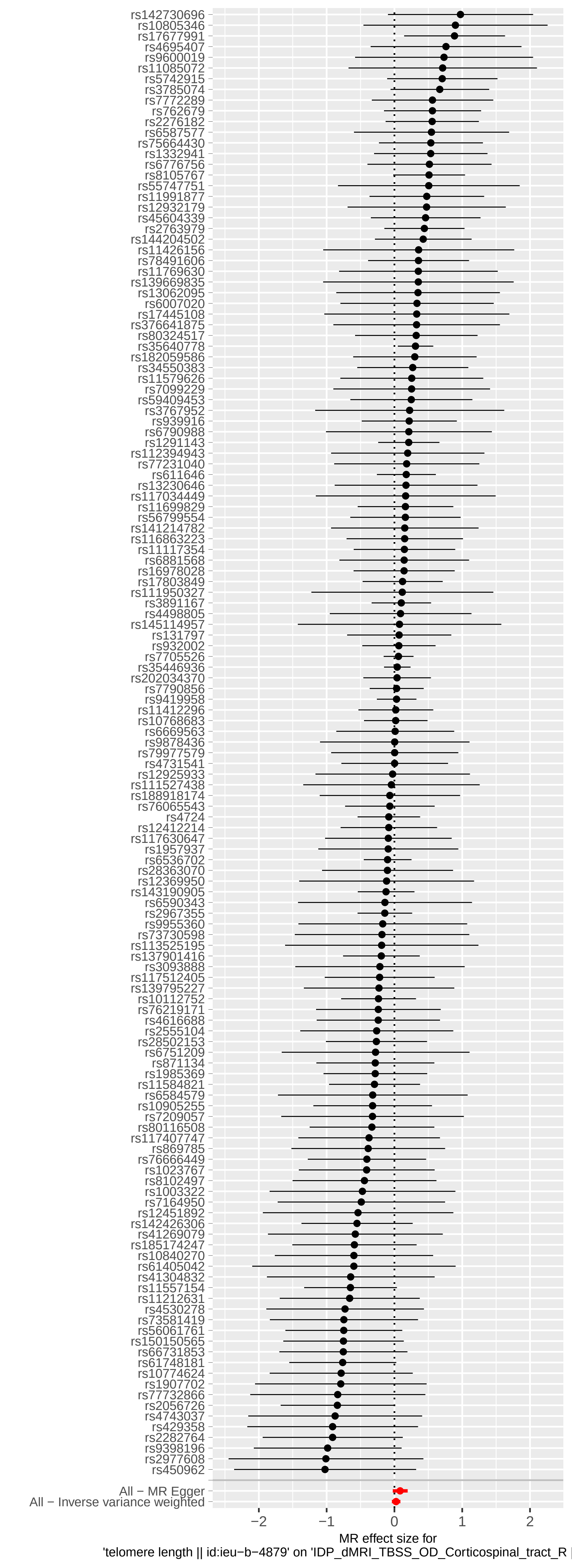
rs142730696
rs10805346
rs17677991
rs4695407
rs9600019
rs11085072
rs5742915
rs3785074
rs7772289
rs762679
rs2276182
rs6587577
rs75664430
rs1332941
rs6776756
rs8105767
rs55747751
rs11991877
rs12932179
rs45604339
rs2763979
rs144204502
rs11426156
rs78491606
rs11769630
rs139669835
rs13062095
rs6007020
rs17445108
rs376641875
rs80324517
rs35640778
rs182059586
rs34550383
rs11579626
rs7099229
rs59409453
rs3767952
rs939916
rs6790988
rs1291143
rs112394943
rs77231040
rs611646
rs13230646
rs117034449
rs11699829
rs56799554
rs141214782
rs116863223
rs11117354
rs6881568
rs16978028
rs17803849
rs111950327
rs3891167
rs4498805
rs145114957
rs131797
rs932002
rs7705526
rs35446936
rs202034370
rs7790856
rs9419958
rs11412296
rs10768683
rs6669563
rs9878436
rs79977579
rs4731541
rs12925933
rs111527438
rs188918174
rs76065543
rs4724
rs12412214
rs117630647
rs1957937
rs6536702
rs28363070
rs12369950
rs143190905
rs6590343
rs2967355
rs9955360
rs73730598
rs113525195
rs137901416
rs3093888
rs117512405
rs139795227
rs10112752
rs76219171
rs4616688
rs2555104
rs28502153
rs6751209
rs871134
rs1985369
rs11584821
rs6584579
rs10905255
rs7209057
rs80116508
rs117407747
rs869785
rs76666449
rs1023767
rs8102497
rs1003322
rs7164950
rs12451892
rs142426306
rs41269079
rs185174247
rs10840270
rs61405042
rs41304832
rs11557154
rs11212631
rs4530278
rs73581419
rs56061761
rs150150565
rs66731853
rs61748181
rs10774624
rs1907702
rs77732866
rs2056726
rs4743037
rs429358
rs2282764
rs9398196
rs2977608
rs450962
All − MR Egger
All − Inverse variance weighted
−2
−1
0
1
2
MR effect size for
'telomere length || id:ieu−b−4879' on 'IDP_dMRI_TBSS_OD_Corticospinal_tract_R |
